# Prevalence of herbal and dietary supplement usage in Thai outpatients with chronic kidney disease: a cross-sectional survey

**DOI:** 10.1186/1472-6882-13-153

**Published:** 2013-07-01

**Authors:** Mayuree Tangkiatkumjai, Helen Boardman, Kearkiat Praditpornsilpa, Dawn M Walker

**Affiliations:** 1 Division of Primary Care, School of Community Health Sciences, University of Nottingham, Nottingham, UK; 2 Division of Social Research in Medicines and Health, School of Pharmacy, University of Nottingham, Nottingham, UK; 3 Division of Nephrology, Department of Medicine, Faculty of Medicine, Chulalongkorn University, Bangkok, Thailand

## Abstract

**Background:**

There are few studies of the prevalence and patterns of herbal and dietary supplement (HDS) use in patients with chronic kidney disease (CKD), although many researchers and health professionals worldwide have raised concern about the potential effects of HDS on patients with renal insufficiency. A survey was conducted to determine: the prevalence and patterns of HDS use in Thai patients with CKD; the demographic factors related to HDS use; the reasons why Thai patients with CKD use HDS; respondent experiences of benefits and adverse effects from HDS; and the association between conventional medication adherence and HDS use.

**Methods:**

This cross-sectional survey recruited patients with CKD attending two teaching hospitals in Thailand. Data were collected via an interview using a semi-structured interview schedule regarding demographics, HDS usage, reasons for HDS use, and respondent experiences of effects from HDS. Conventional medication adherence was measured using the Thai version of 8-Item Morisky Medication Adherence Scale. Descriptive statistics were used to analyse the prevalence and the patterns of HDS use. Chi-square tests and multiple logistic regression were used to determine any associations between HDS use, demographics and conventional medication adherence.

**Results:**

Four hundred and twenty-one eligible patients were recruited. The prevalence of HDS use in the previous 12 months was 45%. There were no demographic differences between HDS users and non-users, except former drinkers were less likely to use HDS, compared with non-drinkers (OR 0.43, 95% CI 0.25-0.75). Those with a medium level of adherence to conventional medication were less likely to use HDS compared with those with a low level of adherence (OR 0.53, 95% CI 0.32-0.87). Maintaining well-being was most common purpose for using HDS (36%). Nearly 18% used HDS, such as holy mushroom, river spiderwort and boesenbergia, to treat kidney disease. The top three most often reported reasons why respondents used HDS were family and friend’s recommendation, followed by expecting to gain benefit from HDS and wanting to try them. Perceived beneficial effects on renal function from HDS were reported by around 10% of HDS users. Among HDS users, seven patients perceived worsening CKD from HDS, such as river spiderwort, kariyat and wheatgrass. Additionally, 72% of respondents did not inform their doctor about their HDS use mainly because their doctor did not ask (46%) or would disapprove of their HDS use (15%).

**Conclusions:**

Around half of the Thai patients with CKD used HDS. Health professionals should be aware of HDS use amongst such patients and enquire about HDS use as a part of standard practice in order to prevent any detrimental effects on kidney function.

## Background

There is widespread use of herbal and dietary supplement (HDS), particularly in Asian countries [1]. The prevalence of HDS use in the general population ranges from 22% to 77% across Asian countries [2-7]. Patients with chronic diseases, such as diabetes, cardiovascular diseases and cancer have been reported as being more likely to use HDS [8] and the prevalence of HDS use in these populations ranges from 32% to 77% in Thailand, the US and Malaysia [9-13]. There are no differences in the demographic characteristics between HDS users and non-users in patients with chronic illnesses, including CKD regarding age, gender, education and smoking status [9,11,14]. This differs from HDS users in the general populations, who are more likely to be elderly, female, and educated to a higher level in both Asian and western countries [5,15,16]. Whilst patients in Asian countries reported family and friend’s recommendations [9,13,17] as the main reason for using either complementary and alternative medicine (CAM) or herbal medicine, amongst patients with chronic diseases in the US they are used to supplement conventional medicines and because patients want to try HDS [18]. Spanner and Duncan state that prevention is the main reason for using HDS amongst patients with CKD [14]. Although populations with long term conditions have a higher prevalence of HDS use, more than half of patients with chronic illnesses reported not informing either their doctor or health care providers about their HDS use [9,12,13,17,18]. In contrast, patients with CKD were more likely to inform their health care providers about their HDS use (55-67%) [14,19].

The use of CAM, including HDS use has been shown to be related to poor adherence to conventional medication [20,21]. However, Cherniack (2011) found no association between CAM use and medication adherence [22]. There are limited and controversial suggestions as to whether HDS use negatively influences conventional medication adherence [20-23] due to depressive symptoms, costs of conventional medications and dis-satisfaction with the doctor-patient relationship in patients with hypertension [20].

Chronic kidney disease (CKD) is a worldwide health problem leading to a decreased quality of life and increased mortality, and is a condition where the kidneys no longer function as they should due to a decrease in kidney mass, development of glomerular hypertension and intratubular proteinuria. It is classified on a scale of 1–5 based on a level of glomerular filtration rate, with 5 being the lowest level of function. There is a high prevalence of stage 3–5 CKD in the Thai population (8.9%) [24], compared to the median worldwide prevalence of CKD (7.2%) [25]. Stage 3–5 CKD, an estimated glomerular filtration rate (eGFR) of less than 60 ml/min, is associated with a high number of complications [26] so these patients may be more vulnerable to any HDS adverse effects. Additionally, it has been documented in many recent studies that several HDS induce adverse renal effects ranging from worsening kidney function to renal failure, these include aristolochic acid, fangchi and L-glutamine [2,4,27-30]. This has led to concerns about detrimental effects of HDS use in patients with CKD. Despite the concerns of practitioners, only a few surveys in western countries have investigated HDS use in patients with CKD [14,19]. Grabe and Garrison (2004) in the US found 29% of patients with CKD used HDS, whilst Spanner and Duncan (2005) in Canada reported 45% used HDS. However, these studies had small populations and did not investigate patient’s experiences of positive and negative effects of HDS on kidney function or the association between HDS use and adherence to conventional medication. Given the concerns about the effect of HDS on renal function and the higher prevalence of HDS use in Asian countries, it is important to establish the extent and patterns of use amongst CKD patients in Asian countries so health care providers can be better informed and advise their patients accordingly.

To promote herb product use amongst Thai residents and to provide appropriate dosage regimens and indications for such products, the Thai Food and Drug Administration has issued guidance for their safe and effective use [31]. Seventy-one items of Thai folk herbal products, both herbal combinations and single herbal medicines, are in the Thai National List of Essential Medicines (2011). Senna, roselle, java tea and folk remedies, such as ‘Ya Hom’ and ‘Ka sai’ are listed in the Thai National List for alleviating common illnesses but are not recommended for patients with renal insufficiency by the Thai Food and Drug Administration. High dose senna can induce nephritis, whilst roselle and java tea can affect electrolyte imbalance in patients with CKD. Ya Hom and Ka Sai contain camphor, which can accumulate in the body and thus result in renal toxicity [31].

The aims of this survey were to determine: the prevalence and patterns of HDS use in Thai patients with CKD; demographic factors related to HDS use, including the association between conventional medication adherence and HDS use; reasons why Thai CKD patients use HDS; and respondent experiences of benefit and adverse effects from HDS.

## Methods

A cross-sectional survey was conducted from January to June 2012 in kidney clinics in two teaching hospitals, King Chulalongkorn Memorial Hospital in Bangkok representing an urban population and HRH Princess Maha Chakri Sirindhorn Medical Center in Nakhon-Nayok province representing a rural population. Both hospitals are in the central part of Thailand, which has the second highest prevalence of CKD in the country [4]. The study was approved by the Institutional Review Board for Research in Human Subjects at Faculty of Medicine, Chulalongkorn University and Srinakharinwirot University, Thailand, and the Medical School Research Ethics Committee of the University of Nottingham, UK.

A nineteen-item semi-structured questionnaire was adapted from previously developed surveys on herbal medicine designed by Kuo et al. [32] with added questions in order to achieve the research outcomes. This questionnaire was piloted to test the understanding of the questions and the validity and test-retest reliability (intraclass correlation coefficient = 0.73) in a CKD population of the Thai version of 8-Item Morisky Medication Adherence Scale (MMAS-8) [33]. With respect to validity, low medication adherence measured by MMAS-8 had a trend towards worsening CKD; however the total number of respondents was not sufficient to estimate the relationship with a standard error of 5%. The final version of questionnaire consisted of four parts, demographic characteristics, HDS use, experiences of benefits and adverse effects from HDS, and the Thai version of MMAS-8, see Additional file 1. Demographic characteristics included age, gender, current address, education, occupation, smoking status and alcohol consumption. Household income was not included as in the pilot study half of the respondents were unwilling to provide this information. HDS usage included types, medical purposes, dosage forms, doses and duration of HDS use, reasons why respondents use HDS, information sources, how they obtained the HDS and the disclosure of HDS use to their doctor.

The prevalence of HDS usage was defined as regular or occasional use during the previous 12 months. HDS were defined as products containing plant-derived material, either raw or processed ingredients, from one or more plants or containing dietary ingredients, such as vitamins, minerals, amino acids and substances, such as, enzymes, organ tissues, glands and metabolites [34,35]. Additionally, this study focused on HDS use for the treatment of illnesses or health promotion rather than consumption for daily food intake or cosmetic purposes and did not include prescribed dietary supplements.

Inclusion criteria were: adult outpatients with CKD diagnosed by a doctor; an eGFR level of less than 60 ml/min calculated by the Thai Modification of Diet in Renal Disease equation [36]; and that the patients attended a kidney clinic at either setting. Patients who had received either dialysis or a kidney transplant were excluded. All patients who met the inclusion criteria were approached by MT and other trained interviewers in order to determine their willingness to participate in this survey. If they consented, a 19-item semi-structured questionnaire with samples of HDS pictures was administered in face-to-face interviews.

Data analysis consisted of simple frequencies with percentages which were used to determine the prevalence of HDS use and descriptive results. Chi-square tests were performed to determine the factors related to HDS use and any associations between HDS use and conventional medication adherence. Multiple logistic regression analyses were undertaken to determine associations between HDS use and demographic characteristics and conventional medication adherence. Tests were 2-tailed, and a p-value < 0.05 was considered statistically significant.

## Results

### Demographic characteristics of respondents

The total number of respondents was 444 of which 23 (5%) were excluded. Receiving dialysis (n=15) was the main reason for exclusion. Four patients (1%) did not give their consent and another four patients were unable to provide information due to illnesses. Respondents had a mean age of 66 years (SD ± 13) and 54% were women. The majority of respondents were retired (68%) and had stage 3 CKD (71%). Comparing demographics between the population in this survey and the Thai general population found that there were no differences regarding gender, education levels, living in urban or rural areas, smoking and drinking status [37]. However, respondents in the present survey were older than the general CKD population (mean ± SD: 56.8 ± 14.5) [24].

The prevalence of HDS use amongst patients with CKD was 45%. Almost all HDS users combined them with their prescribed, conventional medicines (99%). Demographic characteristics of HDS users and non-users are shown in Table 1. Comparisons between HDS users and non-users found that conventional medication adherence differed across the groups with HDS users more likely to have poor medication adherence (*χ*2 = 8.46, *p* = 0.015), other comparisons found no differences between the groups.

**Table 1:** **Comparison of characteristics between HDS users and non**-**users** (**n**=**421**)

**Characteristics**	**HDS user (n**=**189)**	**Non user (n**=**232)**	***χ***^***2 ***^***p***-**value**
Age			0.334
≤ 60	62 (32.8%)	66 (28.4%)	
> 60	127 (67.2%)	166 (71.6%)	
Gender			0.708
Male	89 (47.1%)	105 (45.3%)	
Female	100 (52.9%)	127 (54.7%)	
Education			0.862
Less than secondary school	104 (55.3%)	130 (56.0%)	
Secondary school	27 (14.4%)	40 (17.2%)	
Vocational degree	14 (7.4%)	15 (6.5%)	
Undergraduate degree	34 (18.1%)	35 (15.1%)	
Higher than undergraduate degree	9 (4.8%)	12 (5.2%)	
Address			0.186
Capital city	60 (31.7%)	88 (37.9%)	
Rural areas	129 (68.3%)	144 (62.1%)	
Smoking status			0.812
Never	122 (64.6%)	147 (63.4%)	
Former	59 (31.2%)	72 (31.0%)	
Current	8 (4.2%)	13 (5.6%)	
Alcoholic consumption			0.080
Never	119 (63.3%)	121 (52.4%)	
Former	60 (31.9%)	96 (41.5%)	
Current	9 (4.8%)	14 (6.1%)	
Stages of CKD			0.936
3	133 (70.4%)	164 (70.7%)	
4	49 (25.9%)	58 (25.0%)	
5	7 (3.7%)	10 (4.3%)	
Medication adherence**			0.015*
Low	61 (32.3%)	47 (20.2%)	
Medium	79 (41.8%)	122 (52.6%)	
High	49 (25.9%)	63 (27.2%)	

* Statistically significant at p < 0.05; ** Medication adherence was measured using the Thai version of 8-Item Morisky Medication Adherence Scale; Low, medium and high adherence was defined as MMAS < 6, 6 < MMAS < 8, MMAS = 8, respectively.

Further analysis using multiple logistic regression found that former drinkers (Odds ratio (OR) 0.43, 95% CI 0.25-0.75) and respondents having a medium level of adherence to prescribed, conventional medicines (OR 0.53, 95% CI 0.32-0.87) were less likely to use HDS than non-drinkers and those reporting poor adherence. No other statistically significant factors associated with HDS use were found, see Table 2.

**Table 2:** **Multiple logistic regression analysis of factors related to HDS use in patients with CKD** (**n**=**421**)

**Factors**	**Odds ratio**	**95% Confidence interval**
Age		
≤ 60	1.00	
> 60	0.84	0.52-1.36
Gender		
Male	1.00	
Female	0.77	0.47-1.29
Education		
Less than secondary school	1.00	
Secondary school	0.77	0.43-1.40
Vocational degree	1.11	0.48-2.52
Undergraduate degree	1.16	0.64-2.11
Higher than undergraduate degree	0.88	0.33-2.36
Current address		
Capital city	1.00	
Rural address	1.38	0.90-2.12
Smoking status		
Never smoked	1.00	
Former smoker	1.57	0.85-2.89
Current smoker	0.90	0.33-2.47
Alcohol consumption		
Never	1.00	
Former drinker	0.43	0.25-0.75
Current drinker	0.52	0.20-1.33
Stages of CKD		
3	1.00	
4	1.02	0.64-1.64
5	0.92	0.32-2.63
Prescribed, conventional medication adherence		
Low	1.00	
Medium	0.53	0.32-0.87
High	0.68	0.39-1.20

### Herbal and dietary supplement use

Of the respondents using HDS (n=189), a total of 64% reported using herbal medicines and 36% used dietary supplements, with 14% using both. The mean number of different HDS used was 1.6 (SD ± 0.9) products. Oral capsules or tablets were the usual forms of HDS used (51%) of which 11% were traditional Thai or Chinese pills called ‘Luke Klon’, which is a black round pill. Seven percent of the products used were non-processed herbs. Nearly three quarters of the products were used daily (71%) and half of products had been used for less than a year (52%).

Purposes of using HDS were for maintaining well-being (36%), followed by the treatment of other chronic diseases (24%), minor ailments (19%), and kidney diseases (17%). Herbs tended to be used for illnesses, including other chronic diseases, kidney diseases, minor ailments and leg oedema (80%); whereas most dietary supplements were used for well-being (69%).

Family and friends were an important influencing factor for respondents choosing to use HDS (35%). They provided not only HDS information (52%), but also the HDS products (27%), see Table 3. The next most frequently cited reasons for HDS use were that respondents perceived that they would gain benefits from the HDS (22%) and were willing to try HDS (19%). Television, radio and internet (23%) were reported as the second sources of HDS information, followed by health care providers (5%).

**Table 3:** **Reasons for HDS use and information sources** (**n**=**189**)

	**Frequency**	**Percentage**
Reasons (n=317)*		
Family/friend’s recommendation	111	35.0
HDS will work	71	22.4
Willing to try anything that helps	61	19.2
Prefer to use HDS	34	10.7
Health care provider’s recommendation	21	6.6
Safer than conventional medicines	9	2.8
Easy access	5	1.6
Recommended by traditional practitioners or HDS sellers	2	0.7
Experienced adverse effects from conventional medicines	2	0.7
Recommended by other patients with CKD	1	0.3
Information sources (n=222)*		
Family and friends	115	51.8
TV, radio or internet	50	22.5
Health care providers	12	5.4
Books or newspapers	12	5.4
Traditional practitioners	8	3.6
HDS companies	7	3.1
Leaflets from HDS companies	7	3.1
Own knowledge of HDS	7	3.1
Other patients with CKD	2	1.0
Scientific evidence	2	1.0

* Respondents reported more than one reason and more than one information source, so these total more than 189.

Most HDS products were bought from pharmacies, herbal or dietary supplement shops (41%), followed by direct sale HDS companies (30%) who advertise on satellite television or radio to sell their products. Collecting herbs from their own garden were reported by 9% of respondents. Importantly, most respondents reported that they did not inform their doctor about HDS use (72%) and the main reason for this was that their doctor did not ask them (46%), see Table 4.

**Table 4:** **Disclosure of HDS use to doctors and the reasons for not disclosing** (**n**=**189**)

	**Frequency**	**Percentage**
Informed their doctor about their HDS use		
Yes	53	28.0
No	136	72.0
Reasons for not reporting HDS use (n=144)*		
Health care providers don’t ask	66	45.8
Patients worried that their doctor will disapprove of HDS use	22	15.3
Short-term or occasional use	19	13.2
No need to inform their practitioner	18	12.5
Didn’t see their doctor during the period of HDS use	8	5.5
Just start using HDS and no opportunity	4	2.8
Stopping or planning to stop using HDS	3	2.1
Don’t want to tell them	2	1.4
HDS are safe	1	0.7
Doesn’t influence their disease (s)	1	0.7

* Respondents reported more than one reason, so these total more than 136.

### Types of HDS use and perception of benefits and adverse effects from HDS

Amongst 304 different HDS used, there were 58 different herbal products and 18 different dietary supplement products used. Of the herbal products, 66% were a single herbal use and 34% were a herbal combination. However, 51 HDS products (17%) were unknown as respondents did not either know or remember their ingredients. Kariyat (12%), turmeric (10%) and horseradish tree (8%) were the three were most used herbs, see Table 5. Senna, folk remedies “Ya Hom” and “Ka Sai”, java tea and roselle juice were also reported (6%).

**Table 5:** **Herbs used**; **respondents reported purposes and adverse effects** (**n**=**199**)*

**Types of herb used**	**Frequency (%)**	**Main purpose given by respondents**	**Adverse effects experienced**
Kariyat (*Andrographis paniculata*)	23 (11.6)	Common cold, fever, sore throat, diabetes	Increased SCr
Turmeric (*Curcuma longa*)	19 (9.5)	Gastrointestinal symptoms**, constipation, CKD	-
Horseradish tree (*Moringa oleifera*)	16 (8.0)	Diabetes, hypertension, constipation	Unable to stop bleeding
Mixed botanical extract or fruit drink	12 (6.0)	CKD, diabetes, well-being	-
Ginseng (*Panax spp*.)	7 (3.5)	Well-being	-
Holy mushroom (*Garnoderma lucidum*)	5 (2.5)	CKD	Oedema
River spiderwort (*Tradescantia fluminensis*)	4 (2.0)	CKD	Increased SCr, fatigue
Babbler’s Bill Leaf (*Thunbergia laurifolia*)	3 (1.5)	Detoxification, diabetes	-
Senna (*Senna alexandrina*)	3 (1.5)	Constipation	-
Ginkgo (*Ginkgo biloba*)	3 (1.5)	Improved brain function	-
Boesenbergia (*Boesenbergia rotunda*)	3 (1.5)	CKD	-
Garlic	3 (1.5)	Dyslipidemia	-
Mixed Thai traditional herbs called “Ya Hom”	3 (1.5)	Well-being, fainting, dizziness	-
Heart-leaved moonseed (*Tinospora cripa*)	3 (1.5)	Diabetes,well-being	-
Coix seed (*Semen Coicis*)	3 (1.5)	CKD, well-being, diabetes	-
Vap Ca (*Houttuynia cordata*)	2 (1.0)	Kidney stones, CKD	-
Aloe (*Aloe vera*)	2 (1.0)	Diuretic effects, well-being	-
Blue Pea (*Clitoria ternatea*)	2 (1.0)	CKD	-
Mixed 3 or 6 types of mushrooms	2 (1.0)	CKD	-
Shiitake mushroom (*Lentinus edodes*)	2 (1.0)	CKD, well-being	-
Cinnamon (*Cinnamomum verum*)	2 (1.0)	Diabetes	-
Mixed Thai traditional herbs called “Ya Khom”	2 (1.0)	Fever	-
Mixed Thai traditional herbs called “Ka Sai”	2 (1.0)	Constipation, well-being	-
Jujube (*Zizyphus mauritiana*) and Roselle (*Hibiscus sabdariffa*)	2 (1.0)	CKD, dyslipidemia	-
Spirulina	2 (1.0)	Detoxification, diabetes	-
Lemongrass	1 (0.5)	Dyslipidemia and CKD	-
Boesenbergia, sweet basil, honey and lime juice	1 (0.5)	CKD	Fainting
Boesenbergia, mint, ginger, galangal, lemongrass, kaffir lime leaves and shallots	1 (0.5)	CKD	-
Spring bitter cucumber (M*omordica cochinchinensis*)	1 (0.5)	CKD	-
Lime	1 (0.5)	Kidney stones	-
Chinese folk remedy - Cordyceps, Lovage (*Angelica sinensis*), deer antler velvet, cinnamon and Schisandra berry (*Schisandra chinensis*)	1 (0.5)	CKD	-
Paragrass roots (*Brachiaria mutica*) and pomegranate leaves (*Punica granatum*)	1 (0.5)	CKD	-
Leaves of *Clerodendrum petasites*	1 (0.5)	CKD	-
Java tea	1 (0.5)	Diuretic effects	-

* Respondents reported more than one type of herb used.

** Flatulance, dyspepsia and peptic ulcers; SCr = Serum creatinine.

Vitamins and minerals were the most often reported dietary supplements used (16%, n=17) of which vitamin C (n=6) and calcium supplements (n=4) were most common (Table 6), followed by essence of chicken drink (13%) and germ oil (12%).

**Table 6:** **Dietary supplements used**; **respondents reported purposes and adverse effects** (**n**=**105**)*

**Types of dietary supplement used**	**Frequency (%)**	**Purposes given by respondents**	**Adverse effects experienced**
Vitamins and minerals	17 (16.2)	Well-being	Weight gain
Essence of chicken drink	14 (13.3)	Well-being	Increased blood sugar
Germ oil	13 (12.4)	Well-being	-
Rice Bran oil	9 (8.6)	Well-being, CKD, diabetes	-
Fish oil	8 (7.6)	Well-being, cardiovascular diseases	-
Protein	7 (6.7)	Well-being	Proteinuria
Chlorophyll	6 (5.7)	Well-being, CKD, diabetes, hypertension	-
Swiftlet’s nest drink	5 (4.8)	Well-being	-
Bee pollen	2 (1.9)	Well-being	-
Wheatgrass	2 (1.9)	Well-being	Increased SCr
Fibre	2 (1.9)	Constipation	-
Coconut oil	1 (0.9)	Well-being	Diarrhoea

* Respondents reported more than one type of dietary supplement used; SCr = Serum creatinine.

Regarding perception of benefits from HDS, around three quarters of the HDS used were reported to have benefits (74%). The reported benefits were alleviation of minor ailments, such as constipation (24%), musculoskeletal pain (19%) and flatulence (15%), followed by enhanced well-being (28%) and slowing the progression of their CKD (9%) meaning a decrease in serum creatinine. There were nine different HDS which respondents reported slowing the progression of their CKD: Holy mushrooms (n=3), a herbal combination – Boesenbergia, mint, ginger, galangal, lemongrass, kaffir lime leaves and shallots (n=1), a herbal combination - Boesenbergia, onion, galangal, lemongrass, kaffir lime leaves, lime leaves and mint (n=1), turmeric (n=1), Spring bitter cucumber (n=1), a Chinese folk remedy - Cordyceps, *Angelica sinensis*, Chinese Wolfberry, Astragalus (*Astragalus membranaceus*), *Eucommia ulmoides*, *Codonopsis pilosula* and deer antler velvet (n=1), a herbal combination - Jujube, roselle, boesenbergia and mixed 3 types of mushrooms (n=1), spirulina (n=1) and mangosteen peel juice (n=1).

In contrast, only 10% reported adverse effects from HDS use of which the progression of CKD defined as an increase in serum creatinine or having proteinuria was the most often reported adverse effect (37%), followed by gastrointestinal symptoms (16%) and neurological symptoms (16%). Three products with reported increased serum creatinine were products where the respondents did not know the ingredients.

## Discussion

The population in the current survey (mean ± SD: 66 ± 13) was older than Thai general CKD population (mean ± SD: 56.8 ± 14.5) [24]. However, there was no significant difference comparing with literature on the prevalence of HDS use amongst patients with CKD in Canada (HDS users 64.1±11.6; non-users 60.4±15.0) [14].

The prevalence of HDS use amongst Thai CKD patients was 45%. There were no differences in demographic characteristics between HDS users and non-users in relation to age, gender, smoking status or education. Although this prevalence cannot be directly compared with previous surveys of HDS use in Thailand due to the different definition of HDS use, this prevalence is similar to a survey of HDS use in Thai patients with chronic diseases (45%) [10]. We found the prevalence of HDS use in patients with CKD was greater than the prevalence of herbal use in Thai general population (33%) [4]. The prevalence of the current study is consistent with Spanner and Duncan’s survey (2005) in Canada [14], although their study was using a different population, and they used slightly different definitions of HDS use - defined as current daily intake by Spanner and Duncan.

The current study found a significant association between HDS use and poor adherence to conventional medication. However, there was no relationship between HDS use and a high level of adherence to conventional medication. Reasons for these associations are unknown. Likewise, Krousel-Wood et al. (2010) in the US and Gohar et al. (2008) in the UK found a significant association between the use of CAM and a low level of adherence to conventional medicines amongst patients with hypertension, the association was stronger in African-Americans and women [20,21]. There is no clear pattern of this association and the reason for the association has not been investigated, so further studies are needed before firm conclusions can be made.

Non-drinkers were more likely to use HDS than former drinkers in the current survey. However, there is no literature describing the association between HDS use and drinking in patients with chronic diseases. Our results contrast with US national surveys of CAM users amongst young adults in 2000 where they found former drinkers were more likely to use CAM [38]. This difference could be due to the differing populations especially in relation to age. Further studies are required to examine this association in patients with other chronic diseases.

Treatment of chronic kidney diseases was not the main purpose in using HDS. Almost all patients combined HDS with conventional medicines rather using it as a substitute. There is some consistency with surveys of either CAM or herbal use amongst patients with chronic illnesses in Asian and western countries [9,12,17,18]. All HDS products reported as being used for CKD, except *Astragalus membranaceus*, have no scientific evidence to demonstrate their efficacy from clinical trials. Proteinuria, which indicates worsening kidney function, was reported as an adverse effect of using protein supplements - high protein intake is related to a decrease in renal function [39]. River spiderwort, kariyat and wheatgrass were reported by respondents to increase serum creatinine. However, there is no evidence in the literature to support this and therefore research is needed to determine whether or not such HDS would be harmful or beneficial to patients with CKD.

The Thai National List of Essential Medicines (2011) states that for patients with CKD senna, java tea, roselle, Ya Hom and Ka sai are not recommended [31]. However, we found that some respondents used these products (6%). Given the low number of respondents who reported their HDS use to their doctor, health care providers need to ask patients about such HDS use and explain why it could worsen their condition.

Family and friends influence patient’s decision-making regarding HDS use amongst Asian populations as supported by this study and other studies of either CAM or herbal use in patients with chronic illnesses in Thailand and Malaysia [9,12,13,17]. Wanting to try HDS was an often reported reason for using HDS, which was also been reported in other surveys of CAM use amongst patients with chronic illnesses in both Asian and western countries [12,18].

Several surveys in Thailand, Malaysia and the US have reported that most patients with chronic diseases, other than CKD, did not disclose their HDS use to their conventional health care providers [9,12,17,18,40]. In contrast, patients with CKD in Canada and the US were more likely to inform their doctor about their HDS use (67% and 55%, respectively) than patients with other chronic diseases (33%) [14,19]. However, this contrasts with the current study and should be highlighted that most patients in this study did not inform their doctor about their HDS use (72%). It would seem that an Asian population is less likely to disclose their HDS use to their conventional health professionals, compared with western populations [40]. Most often reported reasons of non-disclosure were that their doctor did not enquire, they may disapprove of their HDS use or they did not need to know about their HDS use, which is consistent with Robinson and McGrail’s systematic review [41].

The findings suggest that conventional health care providers should be aware of the prevalence of HDS use in patients with CKD. They should always ask about their patient’s use of HDS, monitor the impact of HDS on CKD, and be prepared to discuss information about how to safely use HDS products with the patient and their caregivers.

## Conclusions

Around half of Thai patients with CKD used HDS; however most of them did not disclose this use to their conventional health care providers. HDS use was significantly associated with a low level of adherence to conventional medicine. Some products were reported to be used to treat their CKD and were perceived to have beneficial effects on kidney function – despite any robust clinical evidence of efficacy. Moreover, some patients perceived renal adverse effects from HDS, which have never been reported in the literature. These findings should be addressed by conventional health care providers in order to prevent detrimental effects of HDS on patients with CKD. Health care providers should enquire about HDS use in patients with CKD as a part of standard practice. Sellers of HDS should ensure appropriate information about their products safety is available to purchasers. Further studies are required to investigate the efficacy and safety of HDS used by patients with CKD.

## Competing interests

The authors declare that they have no competing interests.

## Authors’ contributions

MT conceived the study, performed the statistical analysis, coordination of the study and drafted the manuscript. KP advised on the feasibility of the study and participated in coordination of the study. MT, DMW and HB designed the study, the questionnaire and data analyses. All authors reviewed the manuscript and have read and approved the final manuscript.

## Pre-publication history

The pre-publication history for this paper can be accessed here:

http://www.biomedcentral.com/1472-6882/13/153/prepub

## Supplementary Material

Additional file 1 **Questionnaire of HDS use.** The questionnaire adapted from Kuo et al. and consisted of demographics, patterns of HDS use, experiences of benefits and adverse effects from HDS, and the 8-item Morisky medication adherence questionnaire. Click here for file
