# Supplementary material for: Prevalence of herbal and dietary supplement usage in Thai outpatients with chronic kidney disease: a cross-sectional survey
Source: BMC Complement Altern Med. 2013 Jul 1;13:153. doi: 10.1186/1472-6882-13-153 (PMC3750602; doi:10.1186/1472-6882-13-153)
Supplement: Additional file 1 — Questionnaire of HDS use. The questionnaire adapted from Kuo et al. and consisted of demographics, patterns of HDS use, experiences of benefits and adverse effects from HDS, and the 8-item Morisky medication adherence questionnaire. [file 1472-6882-13-153-S1.pdf]

Appendix 1

The questionnaire of HDS use

The purpose of this interview is to gather information about the use of herbal and dietary supplements amongst patients with chronic kidney disease (CKD) in order to provide information regarding them for patients with CKD and health care providers.

Participant number……………… Date of data collection………/…………/………….

Demographic data

1. Gender ☐ Male (0) ☐ Female (1)
2. Current address ☐ Bangkok (0) ☐ Rural area (1)
3. Education

   ☐ None (0) ☐ Primary school (1) ☐ Secondary School (2)

   ☐ Vocational degree (3) ☐ Bachelor’s degree (4) ☐ Higher degree (5)
4. Occupation

   ☐ Unemployed (0) ☐ Retired (1) ☐ Housewife (2) ☐ Business owner (3)

   ☐ Employee (4) ☐ Farmer (5) ☐ Professional (6)

   ☐ Other (specify) (7)……………………………..
5. Smoking status

   ☐ Never (0) ☐ Former smoker (1) ☐ Current smoker (2)

   Date stopped……………………… Amount……………..cigarettes, packs/day
6. Alcoholic consumption

   ☐ Never (0) ☐ Former consumer (1) ☐ Current consumer (2)

   Date stopped……………… Types ……………

Amount…………..glasses,cans,bottles per day/week

The use of herbal and dietary supplements

7. Did you use herbal or dietary supplements in the last month?

   ☐ Never (0) (go to question no. 18)

   ☐ Former use (1) Stop date…….…/…………/……...(go to question no.8-16 and 18)

   ☐ Yes (2) (go to question no.8-17):

   ☐ Herbs (0) ☐ Dietary supplements (1) ☐ Both (2)

8. How many herbal and dietary products do you use if any?

   Herbs………………….. Dietary supplements…………………………..

   ☐ Cannot remember (999)

9. What are the products? What purposes do you use them? How do you use them? How much do you use them? How often do you use them? How long do you use them? (please fill in the table)

Name of HDS	Indication[(a)]	Dosage form[(b)]	How do you use[(c)]	Dose (per day)	How often (per day/ week)	How long[(d)] (month/year)

Note: HDS = Herbal and dietary supplements, Missing data = Participants cannot remember (999)

(a) Treatment of CKD (1), Well-being (2), Long life expectancy (3), others (4)

(b) Pills, capsules, tablets, powder, solution, crude herbs

(c) Swallow (1), Topical use (2), Make into a drink/food using hot water (3), other (4)

(d) How long have you used them?

10. Why do you use them? Please rank the top three of reasons (can tick more than 1 answer)

……I wanted to try them (1) ……I hope they will work (2)

……They are safer than modern medicines (3)

……Modern medicines don’t work (4) ……They are cheaper than modern medicines (5)

……They are easily accessed (6) ……Health care providers recommended them (7)

……My family and friends recommended them (8)

……Used by themselves (9)

……Other (specify) (10)…………………………………………………………………

11. From what sources did you receive information about herbal and dietary supplements? (can tick more than 1 answer)

☐ Radio (1) ☐ Television (2) ☐ Newspaper (3) ☐ Leaflet (4)

☐ Internet (5) ☐ Family or friend (6) ☐ Other (specify) (7)………………

12. How did you obtain herbal and dietary supplements? (can tick more than 1 answer)

☐ Buy (1) from ☐ Drug store (1) ☐ Folk remedy shop (2) ☐ Health food store(3)

☐ Hospital (4) ☐ Direct sale (5)

☐ Provided by their family/ friends (2) ☐ Collecting them from their garden (3)

☐ Other (specify) (4)………………………………………………………………

13. Did you inform your doctor that you use them?

☐ No (0) because ☐ They don't ask (1)☐ No need to inform (2)

☐ Other (specify) (3)……………………………………………

☐ Yes (1)

Respondent experiences of benefit and adverse effects from using HDS

14. Have you had any positive effects from herbal and dietary supplements?

☐ No (0) ☐ Yes (1) What are benefits?……………………………………………..

☐ Unsure (2) ☐ Don't know (3)

15. Have you had any problems after taking herbal and dietary supplements?

☐ No (0) (go to question no. 17)

☐ Yes (1) What are problems?…………………………………… (go to question no.16)

Name of HDS…………………………………☐ Unsure (2) ☐ don't know (3)

16. Do you stop using them when you have such problems?

☐ No (1) because………………………………………………………………

☐ Yes (0)

17. How long will you continue to use the HDS? ……………………………months/years

Because…………………………………………………………………………

☐ Unsure (1) ☐ don't know (2)

18. Do you plan to use them within this year? (for participants who have not taken them)

☐ No (0) because……………………………………………………………………………

☐ Yes (1) because…………………………………………………………………………

☐ Unsure (2) ☐ don't know (3)

19. Thai-version of 8-item Morisky medication adherence questionnaire

©Morisky Medication Adherence Scale (MMAS-8-Item). This is a generic adherence scale and the name of the health concern can be substituted in each question item.<br><br>You indicated that you are taking medication for your kidneys. Individuals have identified several issues regarding their medication-taking behaviour and we are interested in your experiences. There is no right or wrong answer. Please answer each question based on your personal experience with your kidney medication.		
**(Please circle the correct number)**		
	**No=1**	**Yes=0**
1. Do you sometimes forget to take your kidney pills?…...		
2. People sometimes miss taking their medications for reasons other than forgetting. Thinking over the past two weeks, were there any days when you did not take your kidney medicine?…...		
3. Have you ever cut back or stopped taking your medication without telling your doctor, because you felt worse when you took it?……...		
4. When you travel or leave home, do you sometimes forget to bring along your kidney medication?………………………...		
5. Did you take your kidney medicine yesterday?………..		
6. When you feel like your kidney disease is under control, do you sometimes stop taking your medicine?………………………………		
7. Taking medication everyday is a real inconvenience for some people. Do you ever feel hassled about sticking to your kidney treatment plan?…………………………………………….		

8. How often do you have difficulty remembering to take all your medications?

(Please circle the correct number)

Never/Rarely……………………….……………..4

Once in a while……………………………………3

Sometimes…………………………….…………..2

Usually……………………………………………1

All the time……………………………..………0
